# Capability and Limitations of Recent Diagnostic Criteria for Autoimmune Pancreatitis

**DOI:** 10.1155/2013/465428

**Published:** 2013-10-30

**Authors:** Taku Tabata, Terumi Kamisawa, Sawako Kuruma, Kazuro Chiba, Susumu Iwasaki, Go Kuwata, Takashi Fujiwara, Hideto Egashira, Satomi Koizumi, Yuka Endo, Koichi Koizumi, Junko Fujiwara, Takeo Arakawa, Kumiko Momma

**Affiliations:** ^1^Department of Internal Medicine, Tokyo Metropolitan Komagome Hospital, 3-18-22 Honkomagome, Bunkyo-ku, Tokyo 113-8677, Japan; ^2^Department of Endoscopy, Tokyo Metropolitan Komagome Hospital, Tokyo 113-8677, Japan

## Abstract

Because a diagnostic serological marker is unavailable, autoimmune pancreatitis (AIP) is diagnosed based on unique features. The diagnostic capabilities and potential limitations of four sets of diagnostic criteria for AIP (Japanese diagnostic criteria 2006 and 2011, Asian diagnostic criteria, and international consensus diagnostic criteria (ICDC)) were compared among 85 patients who were diagnosed AIP according to at least one of the four sets. AIP was diagnosed in 87%, 95%, 95%, and 95% of the patients according to the Japanese 2006, Asian, ICDC, and Japanese 2011 criteria, respectively. The ICDC can diagnose types 1 and 2 AIP independently and show high sensitivity for diagnosis of AIP. However, as the ICDC are rather complex, diagnostic criteria for AIP should perhaps be revised and tailored to each country based on the ICDC.

## 1. Introduction

Autoimmune pancreatitis (AIP) is a specific type of pancreatitis that is thought to have an autoimmune etiology. Since Yoshida et al. [1] proposed AIP as a diagnostic entity in 1995, AIP in various countries, including Japan, has been described. AIP is presently recognized as a pancreatic lesion of IgG4-related disease [2, 3]. Because a diagnostic serological marker is unavailable, it is diagnosed based on unique features. The Japanese diagnostic criteria for AIP were revised in 2006 [4]. The criteria consisted of the following: radiological evidence of pancreatic enlargement and irregular narrowing of the main pancreatic duct; increased serum levels of gammaglobulin, IgG, and IgG4 or the presence of autoantibodies; histological evidence of both lymphoplasmacytic infiltration and fibrosis in the pancreas (Table 1) [4]. 

New diagnostic criteria proposed in Korea [5] and the USA [6] during 2006 included response to steroid therapy and other organ involvement (OOI). The Asian diagnostic criteria that included response to steroids as an optional criterion were published in 2008 (Table 2) [7]. 

AIP comprises histological types 1 and 2 [8, 9]. The histological appearance of type 1 AIP, or traditional AIP, is referred to as lymphoplasmacytic sclerosing pancreatitis (LPSP). Type 2 AIP is histologically characterized by neutrophilic infiltration in the epithelium of the pancreatic duct [8–10]. The international consensus diagnostic criteria (ICDC) for AIP were published for worldwide use and independently diagnose both types of AIP [11]. The criteria comprise five cardinal features, and one or more of which in combination provide the basis for diagnoses of both type 1 and type 2 AIP. The diagnosis of both types can be definitive or probable, or subtypes might not be distinguishable (AIP not otherwise specified (AIP-NOS)) (Table 3) [11].

The ICDC is somewhat complicated for general use, and type 2 AIP is extremely rare in Japan [12, 13]. Based on the Japanese conditions, the Japanese clinical diagnostic criteria for AIP focusing on type 1 AIP were revised in 2011 [14]. They maintained many of the basic concepts of the ICDC, including definite, probable, and possible diagnoses (Table 4) [14].

In this study, based on our experience with AIP, the diagnostic capabilities and potential limitations of these four sets of diagnostic criteria for AIP (Japanese clinical diagnostic criteria 2006 and 2011, Asian diagnostic criteria, and ICDC) were compared.

## 2. Methods

A total of 93 patients who were diagnosed with AIP comprehensively from clinical or radiological findings were treated in Tokyo Metropolitan Komagome Hospital between 1992 and 2012. Four sets of diagnostic criteria among 85 patients who were diagnosed with AIP according to at least one of the four sets of criteria were compared. The 8 patients who improved spontaneously or were followed up conservatively could not be diagnosed with AIP in any of the four diagnostic criteria.

 Twenty-nine patients with pancreatic cancer (mean age, 67.5 years; 20 males and 9 females) were enrolled as a control group. They were diagnosed as having pancreatic cancer clinically and/or histologically.

Statistical analysis was performed using Fisher's exact test. In all test, correct *P* values <0.05 were considered statistically significant.

## 3. Results

Mean age of the 85 AIP patients was 64.1 years. They were 62 males and 23 females. Elevated serum levels of IgG (>1800 mg/dL), IgG4 (>135 mg/dL), and autoantibodies (antinuclear antibody and/or rheumatoid factor) were detected in 46%, 76%, and 45% of the patients, respectively. Diffuse enlargement of the pancreas was revealed on computed tomography/magnetic resonance imaging (CT/MRI) and long or multiple narrowing of the main pancreatic duct was detected on endoscopic retrograde pancreatography (ERP) in 41% and 82% of the patients, respectively. Additionally, other organ involvement (OOI) was detected in 40% (sclerosing cholangitis, *n* = 6; retroperitoneal fibrosis, *n* = 7; swollen salivary/lacrimal glands, *n* = 18; renal involvement, *n* = 4). Histology of the pancreas showing LPSP was gained in 21% of the patients. The histology was highly suggestive according to consensus statement of pathology of IgG4-related disease [15]. All patients who were treated with steroids (73%) responded well.

AIP was diagnosed in 87%, 95%, 95%, and 95% of the patients according to the Japanese 2006, Asian, ICDC, and Japanese 2011 criteria, respectively (Table 5). 

Seven patients who were seronegative, three without ERP, and one without pancreatic enlargement could not be diagnosed based on the 2006 Japanese criteria. Three patients without ERP and one without pancreatic enlargement could not be diagnosed based on the Asian criteria. According to the ICDC, nine patients without elevated serum IgG4 were diagnosed with AIP-NOS, one patient with steroid responsiveness and a level-2 serological criterion was diagnosed with probable type 1 AIP, one patient with steroid responsiveness and ulcerative colitis was diagnosed with probable type 2 AIP, and four patients with one or no level 1 criteria who were not treated with steroids could not be diagnosed. Based on the 2011 Japanese criteria, five patients with segmental pancreatic enlargement and elevated serum IgG4 who were not treated with steroids were diagnosed with probable AIP, 10 patients without elevated serum IgG4 were diagnosed as possible AIP, and two patients with segmental pancreatic enlargement without serological, OOI, or histological criteria that were not treated with steroids, one patient with segmental pancreatic enlargement without ERP, and one patient without pancreatic enlargement could not be diagnosed (Tables 5 and 6).

None of the 29 patients with pancreatic cancer were diagnosed with AIP in any of the four diagnostic criteria.

## 4. Discussion 

Since no diagnostic serological markers are available, AIP must be diagnosed on the presence of unique features. In general, diagnostic criteria should have high sensitivity and specificity and be minimally invasive as well as clinically applicable across a wide range of medical settings. Including a response to steroid therapy among the criteria increases diagnostic sensitivity. However, applying a steroid trial to patients in whom differentiation from malignancy is an issue could result in delayed pancreatic cancer surgery and subsequent cancer progression. Steroid trials should carefully proceed after adequate workup for malignancy generates negative findings.

The diagnostic capabilities and potential limitations of the four sets of diagnostic criteria for AIP [4, 7, 11, 14] were compared. These criteria were generally good at diagnosing AIP. Both the ICDC and Japanese criteria 2011 consist of imaging, serological and histological findings, OOI, and response to steroids. The Japanese criteria 2006 consist of only 3 imaging, serological, and histological findings, which explains why they have the lowest sensitivity. Although OOI is not among the Asian criteria, the sensitivity is similar to that of both Japanese 2011 criteria and the ICDC. Although one patient with OOI was serum IgG4-negative, steroid responsiveness with Asian criteria defined the diagnosis. On the other hand, two serum IgG4-negative patients with autoantibodies were diagnosed with AIP based on the Japanese 2006 and Asian criteria. The specificities of all 4 criteria were 100%.

Types 1 and 2 AIP can be independently diagnosed by the ICDC. Although the Asian criteria aimed to diagnose type 1 AIP, type 2 AIP might also be involved if the patient responds to steroids. Atypical AIP without pancreatic enlargement can be diagnosed only by ICDC, but ruling out pancreatic cancer requires caution. ERP finding of a narrowed main pancreatic duct is mandatory for diagnosing AIP according to the Japanese 2006, Japanese 2011, and Asian criteria. However, the Japanese 2011 criteria do not require ERP to diagnose typical AIP when the pancreas is diffusely enlarged. Furthermore, ERP cannot be applied to some patients with acute pancreatitis, or those who refuse to undergo the procedure, or who have ambiguous pancreatographic findings. In contrast to Japan and Korea, endoscopists in other countries generally avoid injecting the pancreatic ducts of patients with obstructive jaundice for fear of causing pancreatitis. For the ICDC to be applicable worldwide, AIP needs to be diagnosed without ERP. Therefore, ICDC is too complex for general physicians, and since ERP is frequently applied in Japan, the Japanese 2011 criteria might be the most suitable for application in Japan. As the practice of various tests and their perceived accuracy for diagnosing AIP vary considerably worldwide, the diagnostic criteria should be revised and tailored to each country based on the ICDC. On the other hand, as the ICDC and Japanese 2011 criteria place a guard at steroid responsiveness, some patients who are not treated with steroids are difficult to definitively diagnose.

## 5. Conclusion

The ICDC can diagnose types 1 and 2 AIP independently and show high sensitivity for diagnosis of AIP. However, as the ICDC are rather complex, diagnostic criteria for AIP should perhaps be revised and tailored to each country based on the ICDC. 

## Figures and Tables

**Table 1:** Japanese clinical diagnostic criteria of AIP 2006 [4].

(1) Pancreatic enlargement and narrowing of the main pancreatic duct	
(2) Elevation of gammaglobulin, IgG, or IgG4 or presence of autoantibodies	
(3) Histology of LPSP	

Diagnosis: 1 + 2 or 1 + 3.

**Table 2:** Asian diagnostic criteria for AIP [7].

(1) Pancreatic enlargement and narrowing of the pancreatic duct	
(2) Elevation of IgG or IgG4 or presence of autoantibodies	
(3) Histology of LPSP (biopsy)	
(4) Histology of LPSP (resected pancreas)	
(5) Response to steroid	

Diagnosis: 1 + 2, 1 + 3, 1 + 5, or 4.

**Table 3:** International consensus diagnostic criteria for type 1 AIP [11].

(1) (P) Pancreatic enlargement	
Level 1: typical imaging, diffuse	
Level 2: indeterminate imaging, segmental/focal	
(2) (D) Narrowing of the main pancreatic duct on ERP	
Level 1: >1/3 length or multiple stricture	
Level 2: segmental/focal	
(3) (S) Serum IgG4 level	
Level 1: >270 mg/dL	
Level 2: 135–270 mg/dL	
(4) (OOI) Other organ involvement	
Level 1: histology (≥3 criteria), or sclerosing cholangitis, or retroperitoneal fibrosis	
Level 2: histology (2 criteria), or salivary/lacrimal gland swelling, or renal involvement	
(5) (H) Histology of the pancreas (LPSP)	
Level 1: ≥3 of 4 criterions (periductal lymphoplasmacytic infiltrate without granulocytic infiltration, obliterative phlebitis, storiform fibrosis, and abundant IgG4-positive cells)	
Level 2: 2 criteria	
(6) (Rt) Response to steroid	

Definite type 1 diagnosis	
Level 1 histology	
Typical imaging: any non-D level 1/level 2	
Indeterminate imaging: two or more from level 1 + level 2 D	
level 1 S/OOI + Rt or level 1 D + level 2 S/OOI/H + Rt	
Probable type 1 diagnosis	
Indeterminate imaging: level 2 S/OOI/H + Rt	
AIP-NOS	
D1/2 + Rt	

**Table 4:** Japanese clinical diagnostic criteria for AIP 2011 [14].

(1) Pancreatic enlargement	
a: Diffuse type	
b: Segmental/focal type	
(2) Narrowing of the main pancreatic duct on ERP	
(3) Serum IgG4 ≥ 135 mg/dL	
(4) Histology (LPSP)	
a: ≥3 criteria	
b: 2 criteria	
(5) Other organ involvement	
Sclerosing cholangitis, sclerosing dacryoadenitis/sialoadenitis, or retroperitoneal fibrosis	
(6) Effectiveness of steroid therapy	

Definite diagnosis	
4a histology	
Diffuse type	
1a + <3/4b/5>	
Segmental/focal type	
1b + 2 + two or more of <3/4b/5>	
1b + 2 + <3/4b/5> + 6	
Probable diagnosis	
Segmental/focal type	
1b + 2 + <3/4b/5>	
Possible diagnosis	
1 + 2 + 6	

**Table 5:** Diagnosis of AIP according to the four criteria.

	*n* (%)
Japanese diagnostic criteria 2006	
Diagnostic	74 (87%)
Nondiagnostic	11 (13%)
Asian diagnostic criteria	
Diagnostic	81 (95%)
Nondiagnostic	4 (5%)
International consensus diagnostic criteria	
Definitive type 1	70 (92%)
Probable type 1	1 (1%)
AIP-NOS	9 (11%)
Probable type 2	1 (1%)
Nondiagnostic	4 (5%)
Japanese diagnostic criteria 2011	
Definitive	66 (78%)
Probable	5 (6%)
Possible	10 (12%)
Nondiagnostic	4 (5%)

**Table 6:** Cases of discrepancy between four sets of diagnostic criteria.

Case	Japanese 2006	Asian	Japanese 2011	ICDC	Enlargement of pancreas	ERP finding	IgG4 level	OOI	Histology	Steroid responsiveness
1	Unable	Unable	Unable	Definitive	No	Level 1	1160	Level 1	—	Yes
2	Diagnosis	Diagnosis	Possible	AIP-NOS	Level 1	Level 1	34	No	—	Yes
3	Unable	Diagnosis	Possible	AIP-NOS	Level 1	Level 1	11	No	—	Yes
4	Diagnosis	Diagnosis	Possible	AIP-NOS	Level 2	Level 2	39	No	—	Yes
5	Unable	Diagnosis	Possible	AIP-NOS	Level 2	Level 1	35	No	—	Yes
6	Unable	Diagnosis	Definitive	Definitive	Level 1	Level 1	—	Level 1	—	Yes
7	Diagnosis	Diagnosis	Probable	Definitive	Level 2	Level 2	1390	No	—	—
8	Unable	Diagnosis	Definitive	Probable type 1	Level 2	Level 2	12	Level 2	—	Yes
9	Unable	Diagnosis	Possible	AIP-NOS	Level 1	Level 1	—	No	—	Yes
10	Diagnosis	Diagnosis	Probable	Unable	Level 2	Level 2	198	No	—	—
11	Unable	Diagnosis	Possible	AIP-NOS	Level 2	Level 1	—	No	—	Yes
12	Diagnosis	Diagnosis	Probable	Definitive	Level 2	Level 1	571	No	—	—
13	Diagnosis	Diagnosis	Possible	AIP-NOS	Level 2	Level 1	66	No	—	Yes
14	Diagnosis	Diagnosis	Probable	Definitive	Level 2	Level 2	346	No	—	—
15	Unable	Diagnosis	Possible	Probable type 2	Level 2	Level 1	45	No	—	Yes
16	Unable	Unable	Definitive	Definitive	Level 1	—	184	No	LPSP	Yes
17	Diagnosis	Diagnosis	Possible	AIP-NOS	Level 2	Level 1	84	No	—	Yes
18	Diagnosis	Diagnosis	Unable	Unable	Level 2	Level 2	73	No	—	—
19	Diagnosis	Diagnosis	Possible	AIP-NOS	Level 2	Level 1	26.4	No	—	Yes
20	Diagnosis	Diagnosis	Unable	Unable	Level 2	Level 1	34.9	No	—	—
21	Diagnosis	Diagnosis	Probable	Unable	Level 2	Level 1	189	No	—	—
22	Unable	Unable	Definitive	Definitive	Level 1	—	783	Level 2	—	—
23	Unable	Unable	Unable	Definitive	Level 2	—	433	Level 1	—	Yes

## References

[1] Yoshida K, Toki F, Takeuchi T, Watanabe S, Shiratori K, Hayashi N (1995). Chronic pancreatitis caused by an autoimmune abnormality. Proposal of the concept of autoimmune pancreatitis. *Digestive Diseases and Sciences*.

[2] Hardacre JM, Iacobuzio-Donahue CA, Sohn TA (2003). Results of pancreaticoduodenectomy for lymphoplasmacytic sclerosing pancreatitis. *Annals of Surgery*.

[3] Abraham SC, Wilentz RE, Yeo CJ (2003). Pancreaticoduodenectomy (Whipple resections) in patients without malignancy: are they all ‘chronic pancreatitis’?. *The American Journal of Surgical Pathology*.

[4] Okazaki K, Kawa S, Kamisawa T (2006). Clinical diagnostic criteria of autoimmune pancreatitis: revised proposal. *Journal of Gastroenterology*.

[5] Kim K-P, Kim M-H, Kim JC, Lee SS, Seo DW, Lee SK (2006). Diagnostic criteria for autoimmune chronic pancreatitis revisited. *World Journal of Gastroenterology*.

[6] Chari ST, Smyrk TC, Levy MJ (2006). Autoimmune pancreatitis: diagnosis using histology, imaging, serology, other organ involvement and response to steroids. *Clinical Gastroenterology and Hepatology*.

[7] Otsuki M, Chung JB, Okazaki K (2008). Asian diagnostic criteria for autoimmune pancreatitis: consensus of the Japan-Korea symposium on autoimmune pancreatitis. *Journal of Gastroenterology*.

[8] Chari ST, Kloeppel G, Zhang L, Notohara K, Lerch MM, Shimosegawa T (2010). Histopathologic and clinical subtypes of autoimmune pancreatitis: the honolulu consensus document. *Pancreas*.

[9] Sah RP, Chari ST, Pannala R (2010). Differences in clinical profile and relapse rate of type 1 versus type 2 autoimmune pancreatitis. *Gastroenterology*.

[10] Notohara K, Burgart LJ, Yadav D, Chari S, Smyrk TC (2003). Idiopathic chronic pancreatitis with periductal lymphoplasmacytic infiltration: clinicopathologic features of 35 cases. *The American Journal of Surgical Pathology*.

[11] Shimosegawa T, Chari ST, Frulloni L (2011). International consensus diagnostic criteria for autoimmune pancreatitis: guidelines of the international association of pancreatology. *Pancreas*.

[12] Kamisawa T, Chari ST, Giday SA (2011). Clinical profile of autoimmune pancreatitis and its histological subtypes: an international multicenter survey. *Pancreas*.

[13] Hart PA, Kamisawa T, Brugge WR (2012). Long-term outcomes of autoimmune pancreatitis: a multicentre, international analysis. *Gut*.

[14] Shimosegawa T (2012). The amendment of the clinical diagnostic criteria in Japan (JPS2011) in response to the proposal of the international consensus of diagnostic criteria (ICDC) for autoimmune pancreatitis. *Pancreas*.

[15] Deshpande V, Zen Y, Chan JK (2012). Consensus statement on the pathology of IgG4-related disease. *Modern Pathology*.

